# Preventing Post-Traumatic Stress Disorder (PTSD) in rats with pulsed 810 nm laser transcranial phototherapy

**DOI:** 10.1038/s41398-023-02583-3

**Published:** 2023-08-14

**Authors:** Hongyou Zhao, Yi Li, Ting Luo, Wenxin Chou, Tianzhen Sun, Haolin Liu, Haixia Qiu, Dan Zhu, Defu Chen, Ying Gu

**Affiliations:** 1 School of Medical Technology, Beijing Institute of Technology, Beijing, China; 2 Department of Laser Medicine, the First Medical Center of the PLA General Hospital, Beijing, China; 3 Moores Cancer Center, University of California San Diego, San Diego, USA; 4 No.965 Hospital, Joint Logistics Support Force of Chinese PLA, Jilin, China; 5 Britton Chance Center for Biomedical Photonics, Wuhan National Laboratory for Optoelectronics, Huazhong University of Science and Technology, Wuhan, China

**Keywords:** Psychiatric disorders, Neuroscience

## Abstract

Post-traumatic stress disorder (PTSD) is a debilitating condition that occurs following exposure to traumatic events. Current treatments, such as psychological debriefing and pharmacotherapy, often have limited efficacy and may result in unwanted side effects, making early intervention is a more desirable strategy. In this study, we investigated the efficacy of a single dose of pulsed (10 Hz) 810 nm laser-phototherapy (P-PT) as an early intervention for preventing PTSD-like comorbidities in rats induced by single inescapable electric foot shock following the single prolonged stress (SPS&S). As indicated by the results of the open filed test, elevated plus maze test, and contextual fear conditioning test, P-PT prevented the development of anxiety and freezing behaviors in rats exposed to the SPS&S. We also compared the effects of P-PT and continuous wave 810 nm laser-phototherapy (CW-PT) in preventing PTSD-like comorbidities in rats. The results revealed that P-PT was effective in preventing both freezing and anxiety behavior in stressed rats. In contrast, CW-PT only had a preventive effect on freezing behavior but not anxiety. Additionally, P-PT significantly reduced the *c-fos* expression in cingulate cortex area 1(Cg1) and infralimbic cortex (IL) of stressed rats, while CW-PT had no significant effects on *c-fos* expression. Taken together, our results demonstrate that P-PT is a highly effective strategy for preventing the occurrence of PTSD-like comorbidities in rats.

## Introduction

Post-traumatic stress disorder (PTSD) is a mental health condition that can be triggered by exposure to traumatic events, such as combats and natural disasters [1, 2]. Studies suggested that a significant proportion of individuals (ranging from 37 to 92%) experience a traumatic event during their lifetime [3], and approximately 7% of American adults currently have PTSD [4]. Certain population, such as veterans, are at a high-risk of developing PTSD due to the nature of their work. For instance, up to 30% of Vietnam War veterans have been diagnosed with PTSD, and 15% still experience symptoms after 15 years since the war. Among Gulf War veterans, the incidence of PTSD is around 8% [5]. The symptoms of PTSD include avoidance, intrusive symptoms and flashbacks, mood and cognitive disruptions, and hyperarousal/reactivity symptoms [6]. These symptoms can lead to numerous problems for patients, including depression, alcohol and substance abuse, conduct problems, social phobia, panic disorder, and even suicidal thoughts or self-harm [7, 8].

Psychotherapy is a commonly used intervention for PTSD patients and can alleviate the symptoms [9]. However, individual reactions to psychotherapy vary. Some PTSD patients may feel exposure therapy too distressing to tolerate it, while others may even experience secondary trauma [10, 11]. In addition to psychotherapy, pharmacological treatments such as antidepressants, anxiolytics, and selective serotonin reuptake inhibitors (SSRI) can also provide relief from symptoms. However, the long-term use of medication may result in unwanted side effects [12–14]. As a result, it is urgent to explore non-invasive and effective prevention/treatment strategies for individuals with PTSD.

Photobiomodulation (PBM), also known as low level laser therapy (LLLT), was discovered by Endre Mester in 1967. He found that LLLT could enhance hair regrowth and wound healing in tumor-implanted rats [15]. The mechanism of PBM involves the activation of cytochrome c oxidase (CCO) of mitochondria by laser irradiation, leading to increased adenosine triphosphate (ATP) and reactive oxygen species (ROS) production, which improves cell viability and cellular proliferation through activation of several signaling pathways in cells [16–19]. Over the past two decades, transcranial PBM emerged as a promising non-invasive treatment option for various brain disorders, such as stroke, traumatic brain injury (TBI), Alzheimer’s disease (AD), Parkinson’s disease, and psychiatric disorders [20, 21]. Although continuous wave (CW) near-infrared (NIR) light/laser has been commonly used in PBM [22], a study by Takahiro et al. has shown that the therapeutic effect of LLLT for traumatic brain injury with an 810 nm laser was more effective at 10 Hz pulse frequency than at CW and 100-Hz in mice [23]. Anita et al. have also demonstrated positive outcomes in the treatment of patients with dementia and AD using 810 nm, 10 Hz pulsed, light-emitting diodes [24].

PTSD is unique among brain disorders in that it can be directly linked to a causal event for up to one month following the event, which provides an opportunity for early intervention [25]. In a recent study, Yong Li et al. reported that early CW 808 nm laser transcranial irradiation can prevent the occurrence of PTSD-like comorbidities in rats [26, 27]. However, whether pulsed laser transcranial irradiation has an effect on the prevention of PTSD remains unknown. Therefore, the objectives of this study are to investigate the effect of pulsed 810 nm laser transcranial irradiation (10 Hz) on the prevention of PTSD in rats and to compare its therapeutic effect with that of CW 810 nm laser irradiation. Our results showed that a single dose of pulsed 810 nm laser-phototherapy (P-PT), as an early intervention, displayed a better therapeutic effect in the prevention of PTSD-like comorbidities in rats induced by an SPS&S procedure. This is the first study to demonstrate that P-PT is an effective strategy for preventing the occurrence of PTSD-like comorbidities in rats.

## Materials and methods

### Animals

Male Sprague–Dawley rats (7 weeks old, 220–250 g body weight) were obtained from SPF (Beijing) Biotechnology Co., Ltd. The rats were housed individually and had free access to food and water under a 12-h light/12-h dark cycle at 23 ± 2 °C and 55 ± 5% humidity. The rats were assigned to one of four groups with random number method: Control (Ctrl), Stress (Str), Stress + Continuous Wave Phototherapy (Str + CW-PT), and Stress + Pulsed Phototherapy (Str + P-PT). The animal experimental protocol was approved by the Institutional Animal Care and Use Committee (IACUC) of Chinese People’s Liberation Army (PLA) General Hospital, and all the procedures were carried out in accordance with the approved guidelines.

### Single inescapable electric foot shock following the single prolonged stress procedure (SPS&S)

To induce PTSD-like symptoms, the rats underwent a multi-stressor protocol based on a previous study [28]. The protocol consisted of immobilization in a plastic container for 2 hours, followed by forced swimming for 20 minutes in water at 24 ± 1 °C and a depth of 50 cm. The rates were then given a 15-min rest period. After the rest period, they were anesthetized until they lost consciousness and subjected to 10 random electric shocks at 1 mA for 6 s each in a 15-min interval. Following the stressor, the rats were returned to their cages and provided with enough food and water for another week before the behavior evaluation.

### Apparatus and phototherapy

**Fig. 1:**
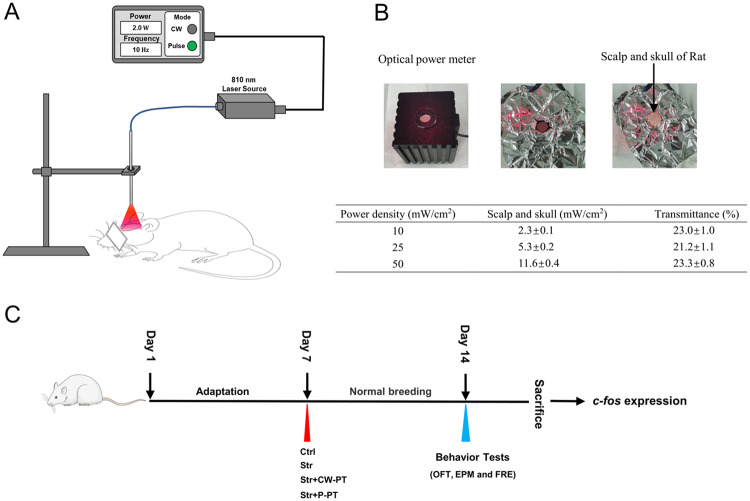
The detailed procedure for applying phototherapy on rats. **A** Rats receiving either continuous wave or pulsed 810 nm laser treatment. **B** Measurement of transmittances of 810 nm laser (Continuous wave) through the scalp and skull of rat (top) and the transmittances of 810-nm laser with different power densities. **C** Timeline of the experimental procedure (Str + CW-PT: Continuous wave-phototherapy after stress, Str + P-PT: Pulsed-phototherapy after stress). Each group, *n* = 6, one-way ANOVA.

The light source for irradiation was a semiconductor laser (810 nm, HOP-100, L.H.H. MEDICAL Tech Co., Ltd., Beijing, China). The study included four groups of rats: Ctrl, Str, Str + CW-PT, Str + P-PT, with 6 rats per group. Rats in the phototherapy groups were anesthetized and received irradiation immediately after SPS&S. The laser output was coupled into an optical fiber (Medlight, S.A. Switzerland) with a lens for an even 3 cm^2^ irradiation area that covered the shaved scalp of rat (Fig. 1A). Both CW and pulsed mode (10 Hz) were applied at a power density of 25 mW/cm^2^ on the rat scalp. The irradiation time lasted 30 min for both phototherapy groups. Rats in the Ctrl and Str groups were sham-operated, but the laser was not turn on.

### Open field test (OFT)

Following a 60-min acclimation period in a room with dim light (15 lux), the rats were placed in the center of a plastic box and allowed to explore the arena for 10 min. The rats’ behavior was monitored and recorded by an infrared camera placed above the box, and the number of entries and duration of stay in the center was measured and analyzed using Any-Maze software.

### Elevated plus maze (EPM) test

After a 60-minute acclimation period to a dark room, the rats were placed on the central platform of the maze, facing the same open arm, and allowed to explore the maze for 10 min. The rats’ behavior was monitored and recorded with an infrared camera positioned above the maze, and the number of entries and duration of stay in the open arm (OA) and closed arm (CA) during the 10 minutes of exploration was measured and analyzed using Any-Maze software. Time in open arm% = (Time in OA)/ [(Time in OA) + (Time in CA)] * 100%.

### Fear conditioning test

Freezing behavior of rat was assessed in the electroshock chamber. The time of rats stayed in the chamber for 6 minutes. The freezing time was measured and analyzed using Freeze frame software for both the full 6-minute period and per minute. The percentage of freezing time was plotted to evaluate the fear level of rat.

### Immunohistochemical staining

The rats were sacrificed in 1–1.5 h after the behavioral tests, and their brain sample were preserved using paraformaldehyde perfusion. The *c-fos* antibody was purchased from Synaptic systems (226003). Immunofluorescence staining with *c-fos* was performed to assess changes in the activation level of neurons in the medial prefrontal cortex (mPFC), including Infralimbic cortex (IL), Prelimbic cortex (PrL), and Cingulate cortex, area 1 (Cg1).

### Statistical analysis

Descriptive statistics, including mean and standard error of the mean, were used to describe the results of behavior tests. The normal distribution and estimate of variation were checked before statistical analysis. Differences between the four groups were compared using one-way ANOVA, with multiple comparisons performed using HSD. A *p*-value < 0.05 was considered statistically significant. To ensure the degree of freedom of ANOVA, the number of rats in each group was set as 6. Statistical analysis was performed using SPSS 26.0 software, and the results were plotted using GraphPad Prism 9.4.0.

## Results

### 810 nm light irradiation apparatus developed for the treatment of PTSD in rats

An 810 nm laser with continuous and pulsed wave was used to irradiate anesthetized rats with a power of 2.0 W and a pulse frequency of 10 Hz (Fig. 1A). The transmittances of continuous 810 nm laser to the scalps and skulls of sacrificed rats were measured using an optical power meter, with transmittances 23 ± 1.0, 21.2 ± 1.1 and 23.3 ± 0.8% obtained at power densities of 10, 25 and 50 mW/cm^2^, respectively (Fig. 1B). The results indicated that the irradiance at the brain was approximately 5 mW/cm^2^ when the scalp irradiance was set at 25 mW/cm^2^. Thus, a power density of 25 mW/cm^2^ was used in the experiments. After 7 days of adaptation, all rats except for the control group, were exposed to SPS&S procedure. The rats in phototherapy groups received 810 nm laser irradiation with continuous or pulse in 30 min of SPS&S. Behavioral tests were performed on all rats in 14 days. Finally, the rats were sacrificed to take the brain tissue for analysis of *c-fos* expression (Fig. 1C).

### P-PT prevented PTSD-like anxiety in OFT

**Fig. 2:**
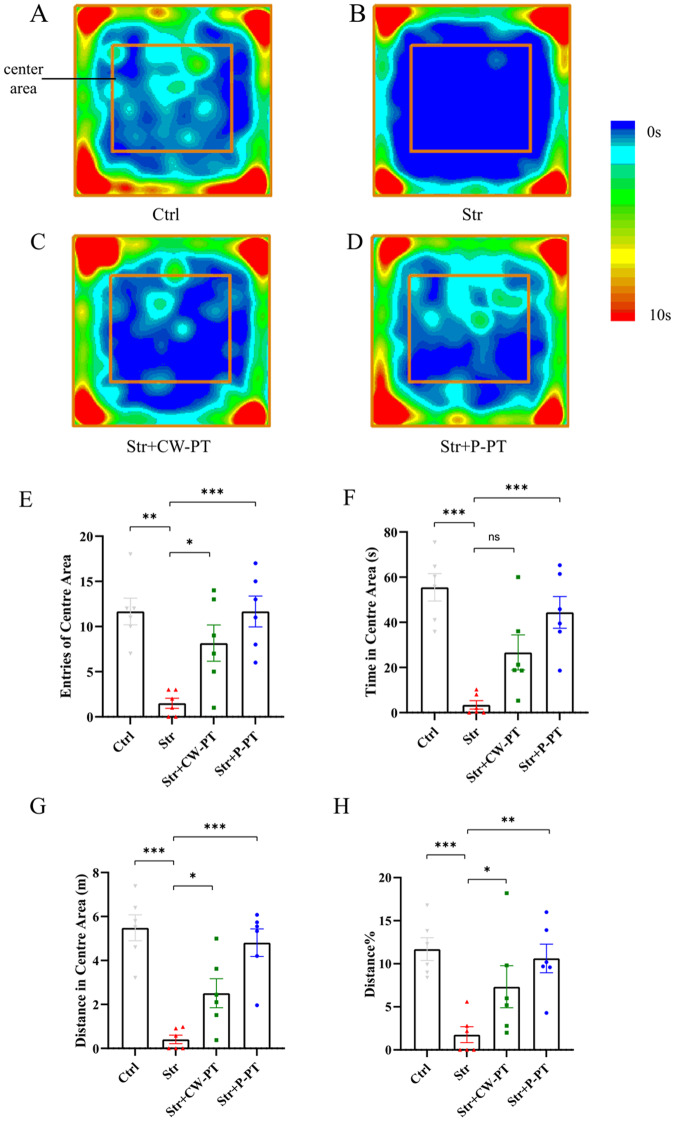
The behaviors of rats in OFT. **A**–**D** Heatmap of the rats’ activities in four groups. **E** Number of entries into the center area. **F** Time spent in the center area. **G** Distance moved in the center area. **H** Rate of moved distance in center area. *P < 0.05, **P < 0.01, ***P < 0.001, ns, no statistical difference. Each group, *n* = 6, one-way ANOVA.

To investigate the effect of transcranial phototherapy on anxiety-like behavior, the open field test, as a classic method, was performed [29]. The heatmap of the rats’ activities in open field are presented in Fig. 2A–D. The rate of moved distance in center area (distance%), which is calculated as (moved distance in center area) / (moved distance in total area), was applied to describe the mobility of rats. Statistical analysis revealed that stressed rats showed less central zone entries, time visiting the center area, moved distance, and the rate of moved distance in center area compared to the Ctrl group (P < 0.01, Str *vs*. Ctrl), indicating serious anxiety (Fig. 2E–H). Stressed rats treated with CW-PT had significantly increased central zone entries, time visiting the center area, moved distance, and rate of moved distance in center area, while those stressed rats treated with P-PT showed the similar behavior to the rats in control group (Fig. 2A and D). Statistical analysis showed no significant difference in rats’ behavior between Str + P-PT and Ctrl group (Fig. 2E and H). These results suggested that P-PT completely prevented the PTSD-like anxiety in rats and CW-PT alleviated the PTSD-like anxiety in rats.

### P-PT but not CW-PT prevented PTSD-like anxiety in EPM test

**Fig. 3:**
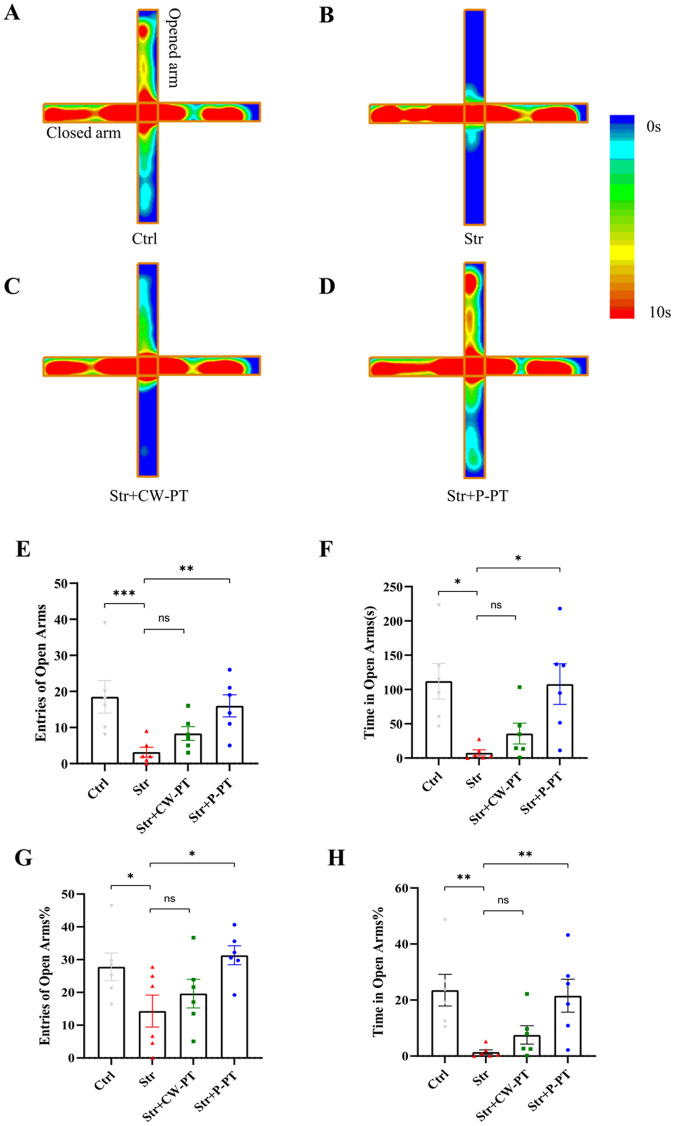
The behaviors of rats in EPM. **A**–**D** Heatmap of the rats’ activities in four groups. **E** Number of entries into open arms. **F** Time spent in open arms. **G** Rates of entries of open arms. **H** Rates of time spent in open arms. *P < 0.05, **P < 0.01, ***P < 0.001, ns: no statistical difference. Each group, *n* = 6, one-way ANOVA.

The elevated plus maze (EPM) test, another classic method to assess the anxiety in rats, was used to further evaluate the effects of transcranial phototherapy on anxiety-like behavior [30]. In the EMP test, the rats’ behavior was assessed by monitoring their activities in the cross, and the results were presented in Fig. 3A–D. The following four statistical measures from the EPM test were analyzed and presented in Fig. 3E–H. Our results showed that the stressed rats had a reduced tendency to enter open arms, while P-PT significantly increased the number of entries (Fig. 3E) and time spent in the open arms (Fig. 3F) in stressed rats. The four statistical measures from the EPM test indicated that there was no significant difference in behaviors between the Str and Str + CW-PT groups, suggesting that CW-PT failed to prevent the occurrence of anxiety. However, there was a significant difference in behaviors of rats between the Str and Str + P-PT groups. More importantly, there was no significant difference in behaviors between Ctrl and Str + P-PT groups (Fig. 3E–H). The results suggested that P-PT prevented the PTSD-like anxiety, but CW-PT was unable to prevent the occurrence of anxiety in rats.

### Both P-PT and CW-PT prevented PTSD-like fear

**Fig. 4:**
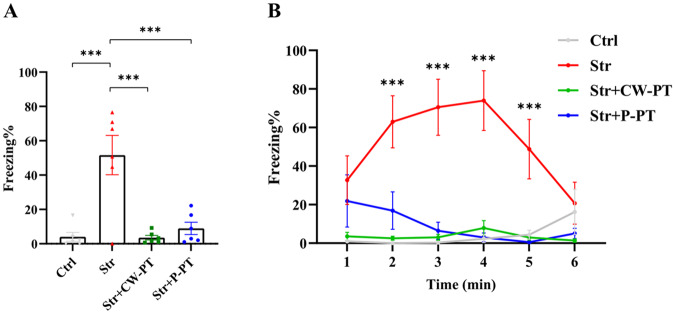
The behaviors of rats in fear conditioning test. **A** The percentage of freezing time in 6 min of the four groups. **B** The percentage of freezing time in each minute of the four groups. *P < 0.05, **P < 0.01, ***P < 0.001. Each group, *n* = 6, one-way ANOVA.

Patients with PTSD usually have an intrusive cued memory (cued hypermnesia) that paradoxically coexists with an impaired contextual memory (contextual amnesia). Therefore, the formation of fear-influenced memories is the one of most important symptoms of PTSD [31]. In our study, stressed rats had significantly higher freezing time compared to the Ctrl group (*P* < 0.001, Str vs. Ctrl), indicating a serious fear-influenced memory. However, rats treated with CW-PT or P-PT had significantly lower freezing time than stressed rats (*P* < 0.001, Str + CW-PT *vs*. Str; *P* < 0.001, STR + P-PT vs. Str). Furthermore, there was no significant difference in freezing time between the phototherapy groups and the Ctrl group (Fig. 4A). We also analyzed the time course of freezing time in four groups and found that CW-PT or P-PT significantly reduced the freezing time of stressed rats at each time point (Fig. 4B). These results suggested that both P-PT and CW-PT prevented the occurrence of PTSD-like fear in rats.

### P-PT but not CW-PT prevented the expression of *c-fos* in the hippocampus and amygdala

**Fig. 5:**
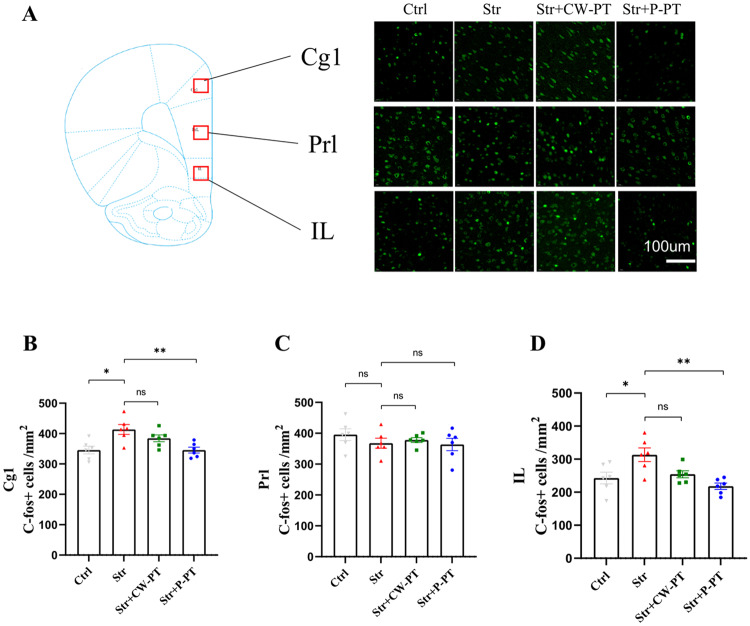
The effect of phototherapy on *c-fos* expression in Cg1, Prl and IL. **A** The areas analyzed for *c-fos* expression in the mPFC (Cg1, Prl, IL). Right: Magnified images show the *c-fos* expression in the boxed area with fluorescence. **B**–**D** Quantitative analysis of *c-fos* expression in Cg1, Prl and IL of the rats in different groups. *P < 0.05, **P < 0.01, ns: no statistical difference. Each group, n = 6, one-way ANOVA.

Hyperactivity in the medial prefrontal cortex (mPFC) is a characteristic of PTSD psychopathology [32], and the expression of *c-fos* is a biomarker of reflecting neural activity [33]. The Fig. 5A showed the expression of *c-fos* in mPFC (Cg1, Prl, IL) of the rats in four groups. The expression of *c-fos* in Cg1 and IL of stressed rats is significantly higher than that in Cg1 and IL of control rats (*P* < 0.05, Fig. 5B, D). There was no significant difference in the expression of *c-fos* in Prl of four groups (P > 0.05, Fig. 5C). P-PT prevented the upregulation of *c-fos* in Cg1 and IL of stressed rats. However, CW-PT did not significantly decrease the *c-fos* expression of stressed rats (Fig. 5B, D). These results suggested that P-PT can prevent the upregulation of *c-fos* in Cg1 and IL induced by SPS&S in rats.

## Discussion

This study explored the preventive effects of pulsed 810 nm laser transcranial irradiation (10 Hz) on PTSD in rats and compared it with that of CW 810 nm laser irradiation. Our results suggest that a single dose of pulsed 810 nm laser transcranial irradiation was able to prevent the development of anxiety and freezing behaviors in rats exposed to the SPS&S. In contrast, CW-PT only had a preventive effect on freezing behavior but not anxiety. Furthermore, P-PT significantly reduced the *c-fos* expression in Cg1 and IL of stressed rats, whereas CW-PT had no significant effects on *c-fos* expression.

Several stimulation methods have been employed to develop rodent animal models that can reflect the symptoms of PTSD patients, including foot shock (FS), underwater trauma (UT), social defeat (SD), single prolonged stress model (SPS), and time-dependent sensitization (TDS) [34] Among of them, the SPS model is widely employed as animal model of PTSD [12]. In this study, we established the SPS&S model by subjecting the rats to electric foot shock immediately after SPS, which enhances the freezing symptom and provides a more accurate evaluation of fear symptoms in subsequent experiments [28]. Our results showed that the stressed rats present obvious PTSD-like anxiety and fear after SPS&S in OFT (*P* < 0.001, Str *vs*. Ctrl) and fear conditioning test (*P* < 0.001, Str *vs*. Ctrl) (Figs. 2 and 3), indicating the successful development of the PTSD rat model.

The mechanism of photobiomodulation (PBM) is widely considered to involve the absorption of photons by mitochondria, resulting in increased activity of cytochrome c oxidase and ATP production, as well as the release of nitric oxide (NO) [34–36]. The efficacy of PBM is determined by several parameters, including wavelength, energy density, laser pulse, and potency [37]. Different lasers with various wavelengths (630 nm, 660 nm, 808 nm and so on) have been used in basic research and clinic. For this study, an 810 nm laser was applied in experiments due to its ability to penetrate skin and skull effectively. Our data shows that over 20 % of the laser power was transmitted through scalp and skull of rat (Fig. 1B). Previous study reported that an irradiance of 25 mW/cm^2^ was applied in experiments since an irradiance of 3–7.5 mW/cm^2^ is known to reach the brain without increasing the temperature during laser irradiation in mice [23]. Transcranial non-invasive PBM is known to allow about 3% of near-infrared photons to penetrate the scalp, bones, and dura mater to reach the surface of the cerebral cortex [38]. In total 808 nm infrared light exhibits a good penetration effect, with an effective depth reaching 4–5 cm below the scalp of human [39].

The therapeutic effect of CW [40, 41] and pulsed [42] laser on diverse neurological diseases has been studied extensively, with pulsed laser showing superior therapeutic outcomes in some cases. For instance, Kymplova et al. demonstrated that a 670-nm pulsed laser at different frequencies (10, 25, and 50 Hz) was more effective in promoting wound healing than a CW light source in a clinical trial [43]. Similarly, Takahiro et al. compared the therapeutic effect of 810 nm CW and pulsed lasers (10 and 100 Hz) on the neurological and histological outcome of the traumatized mice, and found that 10 Hz pulse frequency was the most effective [23]. In our study, we observed that pulsed 810 nm laser (10 Hz) was more effective in preventing PTSD-like comorbidities in rats than CW-PT. P-PT prevented both freezing and anxiety in stressed rats, while CW-PT only prevented freezing (Figs. 2 and 3). These results suggest that the therapeutic effects of P-PT may not be solely attributed to PBM, but also to other yet unknown benefits.

The pulsed frequency of 10 Hz has shown to be effective in some neurological diseases. For instance, Tao et al. reported that 1070 nm light pulsed at 10 Hz could improve cognitive impairment via reducing the cerebral Aβ load in Alzheimer’s disease mouse model [44]. It is possible that the electroencephalogram (EEG) is involved in the therapeutic effect of pulsed laser. The human EEG comprises several frequency bands, including alpha wave (α, 8–13 Hz), beta wave (β, 14–30 Hz), delta wave (δ, 0.5–3.5 Hz), and theta wave (θ, 4–7 Hz) [45]. Resonance may occur between the frequency of the pulsed light and that of the brain waves, particularly in the hippocampal region, where all mammals exhibit prominent theta wave oscillation^23^. Although the neurophysiological etiology of PTSD is unclear, some studies reported that the theta frequency band activity was decreased in both resting and working condition in people with PTSD compared to control subjects [16, 25]. The rats treated with SPS&S showed a significant decrease in delta and theta power spectra have been demonstrated [46]. Our results suggest that 10 Hz (alpha wave) pulsed laser transcranial irradiation prevented the occurrence of PTSD-like comorbidities in rats. Therefore, the low frequency of EEG waves may serve as potential therapeutic targets for PTSD.

The immediate-early gene *c-fos* is a widely used biomarker to reflect the level of brain activation in response to external stimulation [47]. Normally, *c-fos* expression is low, but it is upregulated after external stimulation, peaking around 1.5 h post-stimulation. Consequently, assessing *c-fos* expression after 1.5 h of behavioral testing is an effective method to evaluate brain activation levels during the test [48]. Several studies have reported increased *c-fos* expression in the mPFC of animal models of PTSD [49, 50]. Our study found that the stressed rats had a higher *c-fos* expression in the Cg1 and IL than control rats. P-PT, but not CW-PT, significantly decreased the expression of *c-fos* in the Cg1 and IL of stressed rats. This result suggests that the preventive effect of P-PT on PTSD may be associated with the regulation of *c-fos* expression.

In conclusion, this study demonstrates that a single dose of 10 Hz pulsed 810 nm laser-phototherapy (P-PT) can prevent the occurrence of PTSD-like comorbidities in rats. Both P-PT and CW-PT are effective in the prevention of PTSD-like fear, but P-PT has a better effect in preventing anxiety in stressed rats. More importantly, these results suggest that P-PT (10 Hz, 810 nm) has the potential to be a non-invasive early intervention treatment for preventing the occurrence of PTSD-like comorbidities in humans.
